# Inhibition of Toll-like receptor 4 and Interleukin-1 receptor prevent SARS-CoV-2 mediated kidney injury

**DOI:** 10.1038/s41420-023-01584-x

**Published:** 2023-08-10

**Authors:** Daigo Nakazawa, Yohei Takeda, Masatoshi Kanda, Utano Tomaru, Haruko Ogawa, Takashi Kudo, Satoka Shiratori-Aso, Kanako Watanabe-Kusunoki, Yusho Ueda, Atsuko Miyoshi, Fumihiko Hattanda, Saori Nishio, Ryo Uozumi, Akihiro Ishizu, Tatsuya Atsumi

**Affiliations:** 1 Department of Rheumatology, Endocrinology, and Nephrology, Faculty of Medicine and Graduate School of Medicine, Hokkaido University, Sapporo, Japan; 2 Research Center for Global Agromedicine, Obihiro University of Agriculture and Veterinary Medicine, Obihiro, Japan; 3 Department of Veterinary Medicine, Obihiro University of Agriculture and Veterinary Medicine, Obihiro, Japan; 4 Department of Rheumatology and Clinical Immunology, Sapporo Medical University, Sapporo, Japan; 5 Department of Pathology, Faculty of Medicine and Graduate School of Medicine, Hokkaido University, Sapporo, Japan; 6 Division of Laboratory and Transfusion Medicine, Hokkaido University Hospital, Sapporo, Japan; 7 Department of Medical Laboratory Science, Faculty of Health Sciences, Hokkaido University, Sapporo, Japan

**Keywords:** Antimicrobial responses, Acute kidney injury, Cell death and immune response

## Abstract

Acute kidney injury (AKI) is a common and severe complication of the coronavirus disease 2019 (COVID-19). Severe acute respiratory syndrome coronavirus 2 (SARS-CoV-2) directly affects the glomerular and tubular epithelial cells to induce AKI; however, its pathophysiology remains unclear. Here, we explored the underlying mechanisms and therapeutic targets of renal involvement in COVID-19. We developed an in vitro human kidney cellular model, including immortalized tubular epithelial and endothelial cell lines, demonstrating that SARS-CoV-2 directly triggers cell death. To identify the molecular targets in the process of SARS-CoV-2-mediated cell injury, we performed transcriptional analysis using RNA sequencing. Tubular epithelial cells were more prone to dying by SARS-CoV-2 than endothelial cells; however, SARS-CoV-2 did not replicate in renal cells, distinct from VeroE6/transmembrane protease serine 2 cells. Transcriptomic analysis revealed increased inflammatory and immune-related gene expression levels in renal cells incubated with SARS-CoV-2. Toll-like receptor (TLR) 3 in renal cells recognized viral RNA and underwent cell death. Furthermore, analysis of upstream regulators identified several key transcriptional regulators. Among them, inhibition of the interleukin-1 receptor (IL-1R) and TLR4 pathways protects tubular epithelial and endothelial cells from injury via regulation of the signal transducer and activator of transcription protein-3/nuclear factor-kB pathway. Our results reveal that SARS-CoV-2 directly injures renal cells via the proinflammatory response without viral replication, and that IL-1R and TLR4 may be used as therapeutic targets for SARS-CoV-2 mediated kidney injury.

## Introduction

Patients with severe coronavirus disease 2019 (COVID-19), caused by severe acute respiratory syndrome coronavirus 2 (SARS-CoV-2), develop acute respiratory distress syndrome (ARDS) and multiple organ dysfunction, including the kidneys [1]. COVID-19 is often complicated by fatal acute kidney injury (AKI), particularly among patients requiring intensive care treatment. Direct viral attack, excessive immune responses, drugs, coagulopathies, and other factors may be involved in the pathogenesis of COVID-19-associated AKI (COVID-19 AKI) [2]. Histological studies in autopsy have shown that acute tubular injury is the most common feature in the kidneys of patients with COVID-19 AKI [3]. In some studies, viral RNA and proteins were detected in the kidneys via in situ hybridization and microscopic analysis [4], and infectious SARS-CoV-2 was isolated from urine samples [5]. In contrast, other studies reported that immunostaining for SARS-CoV-2 nucleocapsid (N) protein and RNA in situ hybridization were negative for all tested kidney biopsy samples from patients with COVID-19 AKI [6]. Due to these controversies, it remains unclear how SARS-CoV-2 influences renal cells to initiate AKI. Angiotensin-converting enzyme 2 (ACE2), CD147, and kidney injury molecule 1 (Kim1) are considered to be the receptors of SARS-CoV-2 for entry into renal cells, particularly renal tubules [7, 8]. Transmembrane protease serine 2 (TMPRSS2), an enzyme that proteolytically cleaves S protein of SARS-CoV-2 to activate the binding to cells, is expressed on renal cells and is essential for viral infiltration into the cell [9]. Genetic and environmental variations in these molecules may contribute to the development and susceptibility of AKI [3]. However, the precise mechanism of the development of COVID-19 AKI remains unclear.

Noteworthy is the fact that excessive immune response plays a pivotal role in severe COVID-19. The interaction between virus and host cells results in the induction of proinflammatory responses and organ damage. Toll-like receptors (TLRs) are the innate immune sensors against invading pathogens and are known to be expressed on renal cells. Recent studies have shown that the binding of the RNAs of SARS-CoV-2 with the human TLRs might contribute to the cytokine storm through the activation of immune cells [10, 11].

In the present study, we elucidated the pathogenesis of SARS-CoV-2-mediated kidney injury by evaluating the molecular mechanisms and transcriptional profiles of renal cells in response to SARS-CoV-2. We found that SARS-CoV-2 directly injures renal tubular epithelial cells via TLR4/3 and IL-1R signaling.

## Results

### Morphological change in HK2 and HUEhT cells incubated with SARS-CoV-2

**Fig. 1:**
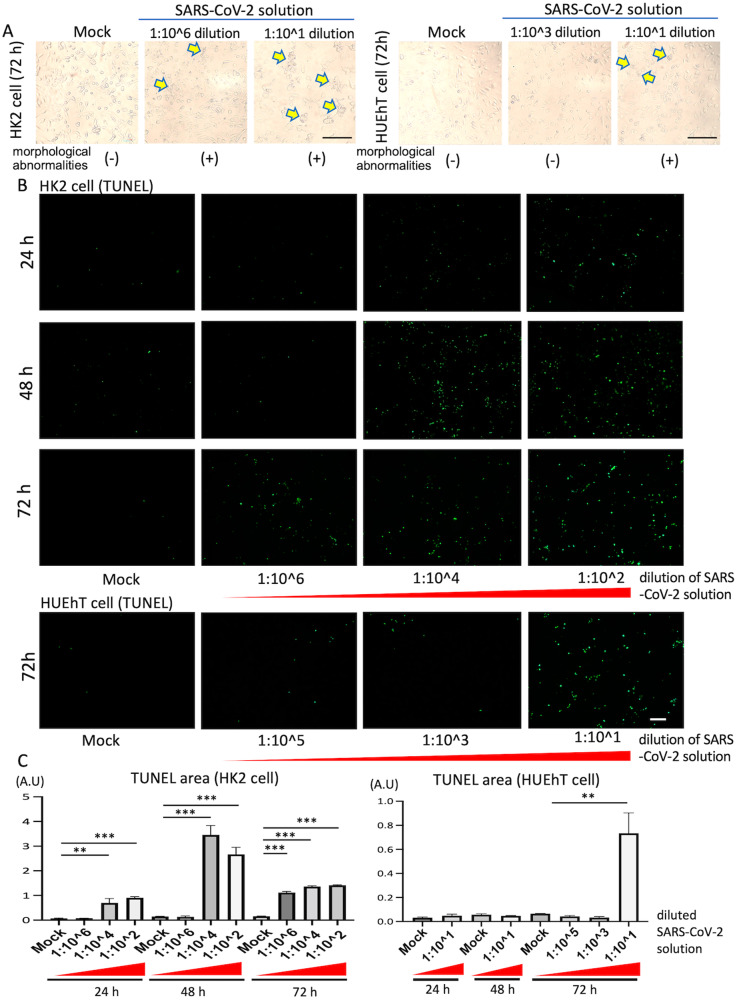
SARS-CoV-2 solution injures HK2 cells and HUEhT cells. **A** Abnormal cell morphologies in HK2 and HUEhT cells were detected at a 1:10^6^ and 1:10^1^ dilution of SARS-CoV-2 solution 72 h after co-culture. Yellow arrowheads show cells with morphological abnormalities. The scale bar is 250 μm. **B** The representative images of TUNEL assay in HK2 and HUEhT cells incubated with SARS-CoV-2 solutions with different dilution ratios. SARS-CoV-2 significantly increased cell death of HK2 cells from culture 24–72 h in a time- and dose-dependent manner. A SARS-CoV-2 with low dilution rate induced cell death of HUEhT 72 h after co-culture. The scale bar is 200 μm. **C** The positive area of TUNEL staining was quantified using the ImageJ software and data are presented as mean ± SEM (each group; *n* = 3). One-way ANOVA with Dunnett’s multiple comparisons tests (control; Mock) was performed for statistical analyses, and significance was defined as **P < 0.01, ***P < 0.001.

In vitro lung models, SARS-CoV-2 infects epithelial cells and induces cell death with inflammatory response [12]. To validate the response of renal cells against SARS-CoV-2, HK2 and HUEhT cells were incubated with different dilutions of SARS-CoV-2 solution for 24–72 h in vitro. Microscopic analysis revealed that cell morphological abnormalities in HK2 and HUEhT cells were detected at 1:10^6^ and 1:10^1^ dilutions of SARS-CoV-2 solution (viral titer: 7 log_10_ 50% tissue culture infective dose [TCID_50_]/mL) 72 h after co-culture, respectively (Fig. 1A). The TdT-mediated dUTP-biotin nick end labeling (TUNEL) assay, which detects dead cells such as necrotic and apoptotic cells [13], revealed that HK2 cells incubated with SARS-CoV-2 underwent cell death from 24–72 h in a time- and dose-dependent manner. Meanwhile, SARS-CoV-2 influenced HUEhT cell at higher concentrations, but did not increase cell death up to 48 h (Fig. 1B, C), indicating that SARS-CoV-2 injures HK2 cells (tubular epithelial cells) severely compared to HUEhT cells (endothelium).

### Infectious and proliferative capacities of SARS-CoV-2 in HK2 and HUEhT cells

**Fig. 2:**
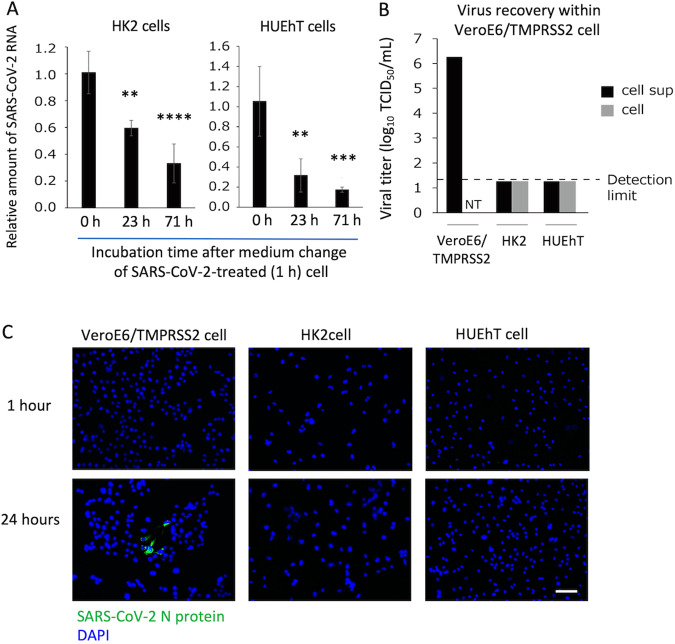
Infectious and proliferative capabilities of SARS-CoV-2 in HK2 and HUEhT cells. **A** Relative amounts of SARS-CoV-2 RNA within HK2 and HUEhT cells 0–71 h after incubation of SARS-CoV-2 solution. The relative amount of viral RNA was quantified by real-time RT-PCR (each group, *n* = 4). One-way ANOVA with Dunnett’s multiple comparisons tests (control; 0 h) was performed for statistical analyses, and significance was defined as **P < 0.01, ***P < 0.001, ****P < 0.0001. **B** Virus recovery within VeroE6/TMPRSS2 cell. Cytopathic effects were evaluated in VeroE6/TMPRSS2 cells in response to supernatants or cell lysates of VeroE6/TMPRSS2, HK2, and HUEhT cells incubated with SARS-CoV-2. **C** Immunostaining of SARS-CoV-2 N proteins in SARS-CoV-2-inoculated-VeroE6/TMPRSS2 cells, HK2 cells, and HUEhT cells (1, 24 h after incubation). Green; SARS-CoV-2 N protein. Blue; DAPI. The scale bar is 100 μm.

Next, to examine whether SARS-CoV-2 infects and replicates within HK2 and HUEhT cells, we measured the temporal change of relative amount of viral RNA in HK2 and HUEhT cells incubated with SARS-CoV-2. In these incubated cells, the medium was changed 1 h after the co-culture with SARS-CoV-2. The relative amount of SARS-CoV-2 RNA, quantified by real-time RT-PCR in HK2 and HUEhT cells, decreased over time (Fig. 2A). Furthermore, we examined virus recovery from the cell culture supernatants and cell lysates of HK2 or HUEhT cells incubated with SARS-CoV-2. The supernatant from VeroE6/TMPRSS2 cells incubated with SARS-CoV-2, which is highly susceptible to SARS-CoV-2, was used as the positive control. Although the supernatant of VeroE6/TMPRSS2 cells incubated with SARS-CoV-2 affected VeroE6/TMPRSS2 cells to induce cytopathic effect (CPE), which is a morphological change of host cells induced by virus infection, the supernatants and cell lysates of HK2 and HUEhT cells incubated with SARS-CoV-2 did not induce CPE on VeroE6/TMPRSS2 cells (Fig. 2B). Immunostaining revealed that SARS-CoV-2 N proteins were detected in the SARS-CoV-2-inoculated VeroE6/TMPRSS2 cells (at 24 h), but these were not detected in SARS-CoV-2-inoculated HK2 and HUEhT cells (Fig. 2C). These findings suggest that SARS-CoV-2 does not replicate nor proliferate within renal cells. Thus, SARS-CoV-2-induced renal cell injury develops independently of SARS-CoV-2 replication in the cells.

### Transcriptional profile of HK2 and HUEhT cells incubated with SARS-CoV-2

**Fig. 3:**
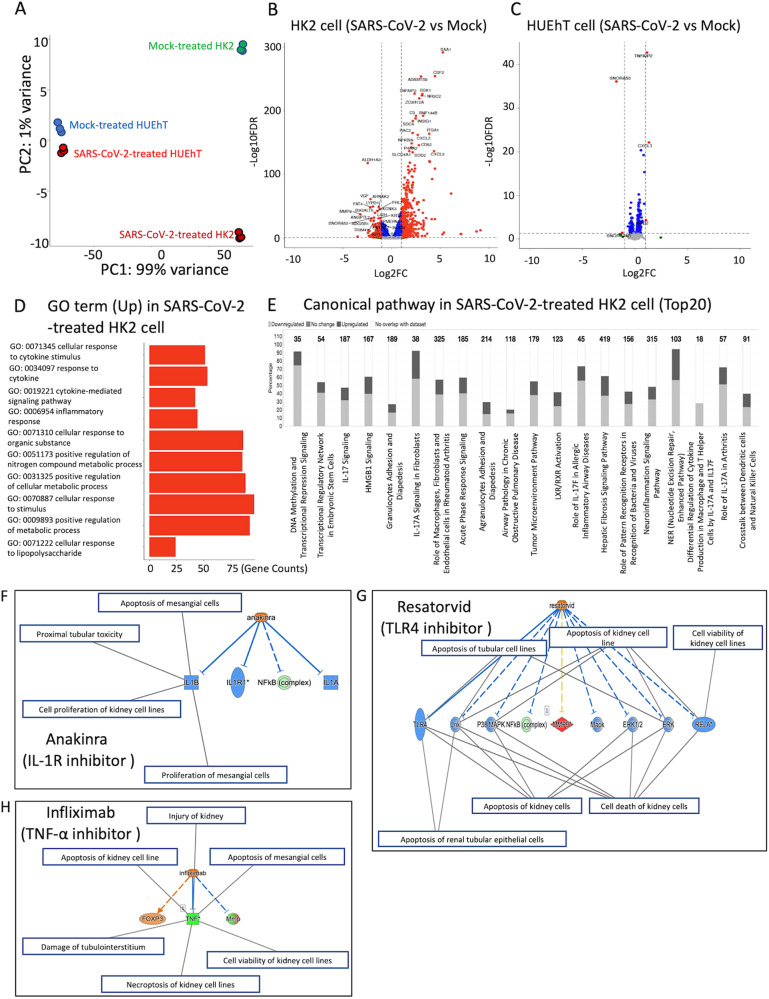
Volcano plot of differential gene expression between HK2 and HUEhT cells incubated with SARS-CoV-2 or mock. **A** RNA-seq was performed on HK2 and HUEhT cells incubated with SARS-CoV-2 or mock. The figure shows the principal component analysis. **B**, **C** Volcano plots of gene expression between cells incubated with SARS-CoV-2 and mock (B; HK2 cells, C; HUEhT cells) were visualized. Protein-cording genes that pass the thresholds for false discovery rate (FDR) and log fold change (FC) (FDR < 0.05 and |log2 FC| > 1) were plotted in red. The characteristic upregulated or downregulated genes for cells incubated with SARS-CoV-2 were mapped. **D** The top 10 adjusted *p*-value GO biological process terms corresponded to upregulated coding gene function in RNA sequencing data of HK2 cells incubated with SARS-CoV-2 compared to HK2 cells incubated with Mock. **E**. Canonical pathway identified by IPA (Top20). **F**–**H** Upstream regulator analysis indicates the relationships between each inhibitor and related genes/renal diseases.

To investigate the mechanism by which SARS-CoV-2 injures renal cells independent of the viral replication, transcriptome profiling was performed via RNA sequencing of HK2 and HUEhT cells incubated with SARS-CoV-2 and mock (each group, *n* = 3). Principal component analysis revealed that HK2 and HUEhT cells incubated with mock had transcriptional profiles distinct from those of HK2 and HUEhT cells incubated with SARS-CoV-2 (Fig. 3A). 834 genes were differentially regulated between the HK2 cells incubated with Mock and those incubated with SARS-CoV-2. Among these, 540 genes were transcriptionally upregulated and 294 were downregulated in HK2 cells incubated with SARS-CoV-2 compared to HK2 cells incubated with Mock (Fig. 3B). Meanwhile, a few differentially expressed genes (DEGs) were detected between the HUEhT cells incubated with Mock and the ones incubated with SARS-CoV-2 (Fig. 3C). Gene ontology (GO) analysis of the transcriptome of HK2 cells incubated with SARS-CoV-2 showed the enrichment of genes related to the activation of the immune system, including cellular responses to cytokine stimuli and inflammatory responses (Fig. 3D). Meanwhile, no significant GO term enrichment was detected in HUEhT cells incubated with SARS-CoV-2, probably because SARS-CoV-2 has little effect on HUEhT cells. Thus, we explored the target molecules for regulating SARS-CoV-2-induced HK2 cell injury using IPA software. IPA revealed that DEGs were significantly enriched in 127 canonical pathways, including inflammation-related pathways, in the HK2 cells incubated with Mock versus SARS-CoV-2 (the top 20 enriched pathways are shown in Fig. 3E). Furthermore, upstream regulator analysis predicted TLR3/4 ligands, interleukin-1 (IL1)B/1A, and TNF for SARS-CoV-2-mediated cell injury (top 50 of upstream regulators are shown in Supplementary Table S1A) and causal network analysis identified the inhibitors, anakinra (IL-1R antagonist), resatorvid (TLR4 inhibitor), and infliximab (TNF-α inhibitor), as master regulators (top 50 of predicted regulators are shown in Supplementary Table S1B). These inhibitors were involved in the mechanistic network of renal cell injury or death via the nuclear factor-kappa B (NF-κB) pathway and TNF signaling (Fig. 3F–H). Similarly, we performed RNA-seq analysis of HUEhT cells incubated with SARS-CoV-2 using IPA and identified resatorvid (Activation z- score; 1.941, a *p*-value of overlap; 0.0894) and anakinra (Activation z- score; 1.948, a *p*-value of overlap; 0.009) as upstream regulators, suggesting that these may be comprehensive therapeutics for SARS-CoV-2 mediated kidney injury. The gene expressions of the key molecules (*IL1B, IL1R1*, *TLR3*, and *TNF*) for the enriched signaling pathways were highly upregulated in HK2 cells compared to those in HUEhT cells (Supplementary Fig. S1A). These might be related to the differences in each cell response.

### Efficacy of therapeutic targets identified by IPA for SARS-CoV-2-induced renal cell injury

**Fig. 4:**
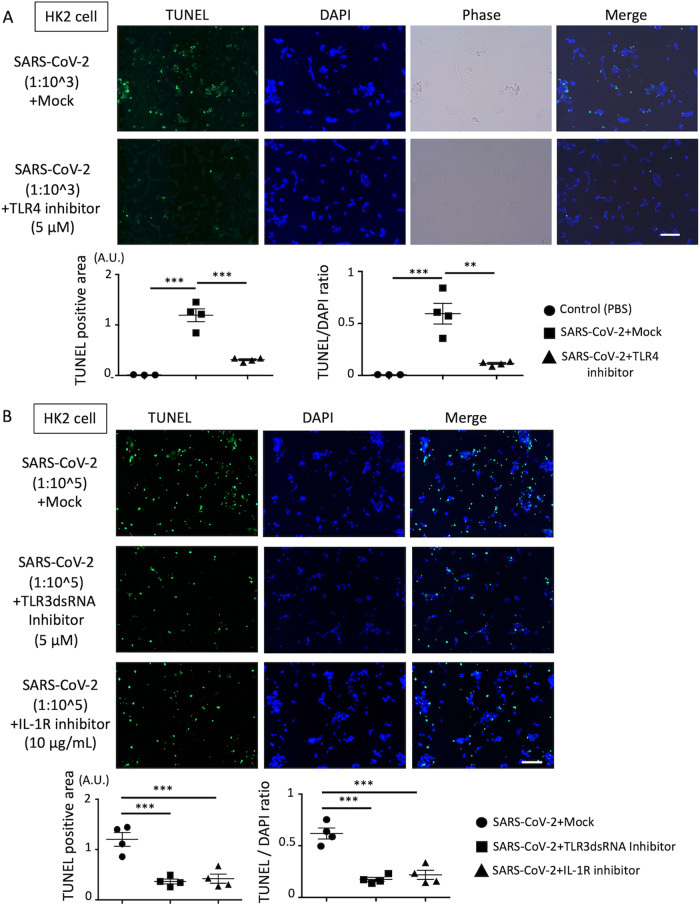
TUNEL staining in HK2 cells incubated with SARS-CoV-2 treated with target inhibitor. **A** HK2 cells incubated with SARS-CoV-2 (dilution/1:10^3^) treated with mock or TLR4 inhibitor (for 72 h incubation). The lower graphs show TUNEL positive area (left) and TUNEL/DAPI ratio (right). **B** HK2 cells incubated with SARS-CoV-2 (dilution/1:10^5^) treated with mock or TLR3dsRNA inhibitor or IL-1R antagonist (for 72 h incubation). The lower graphs show TUNEL positive area (left) and TUNEL/DAPI ratio (right). The lower graphs show TUNEL positive area (left) and TUNEL/DAPI ratio (right). TUNEL/Green, DAPI/Blue. One-way ANOVA with post hoc Tukey’s test was performed for statistical analyses, and significance was defined as **P < 0.01, ***P < 0.001. The scale bar is 100 μm.

We examined whether inhibition of TLR4, TLR3, IL-1R, and TNF signaling identified by IPA ameliorates SARS-CoV-2-induced renal cell injury. The cytotoxicity of these inhibitors in HK2 and HUEhT cells was determined using Cell Counting Kit8 (Supplementary Fig. S1B). First, we assessed the suitable dose of these drugs for cells incubated with SARS-CoV-2 by observing cell morphological changes (48 and 72 h). TLR4 inhibitor affected HK2 cell injury incubated with highly concentrated SARS-CoV-2 solution (dilution/ 1:10^2^) 72 h after incubation. The TLR3dsRNA inhibitor and IL-1R antagonist ameliorated HK2 cell injury when incubated with a low concentration of SARS-CoV-2 solution at 72 h (Supplementary Fig. 2A). The TNF inhibitor did not protect against cell injury under any condition. Furthermore, optical microscopic analysis showed that none of the inhibitors influenced the morphology of HUEhT cells (Supplementary Fig. S2B). Next, we evaluated the efficacy of the TLR4 inhibitor, TLR3dsRNA inhibitor, and IL-1R antagonist by TUNEL staining. TLR4 inhibitor markedly reduced TUNEL-positive cells in a high dose of SARS-CoV-2 (1:10^3^) incubation in HK2 cells. TLR3dsRNA inhibitor and IL-1R antagonist ameliorated HK2 cell injury in a low dose of SARS-CoV-2 (1:10^5^) (Fig. 4A, B). Next, we elucidated how SARS-CoV-2 (an ssRNA virus) influences TLR3 in these cells. We examined TLR3 expression on the cell surface because experimental studies have shown that TLR3 exists on the cell surface as well as endosomes in various cells including HUVEC and might be capable of triggering an immune response [14, 15]. Flow cytometric analysis revealed that a part of TLR3 was detected on the HK2 and HUEhT cell surface, and the expression in HK2 cell was higher than that in HUEhT cell (Supplementary Fig. S2C), indicating that these cells, particularly HK2 cells, might be affected by viral ssRNA via TLR3. In addition, TUNEL staining showed that only IL-1R antagonist protected HUEhT cell injury incubated with a high dose of SARS-CoV-2 (1:10^2^) (Supplementary Fig. S3). These findings suggest that the TLR4 inhibitor has the most significant on protecting against HK2 cell injury and that IL-1R antagonist influences both HK2 and HUEhT cells. Because TLR4 and TLR3 sense cell-derived damage-associated molecular patterns (DAMPs), including high mobility group box 1 (HMGB1), dsRNA, and dsDNA. We needed to exclude the effect of DAMPs contaminating the SARS-CoV-2 solution, which were isolated from the supernatant of SARS-CoV-2-infected VeroE6/TMPRSS2 cells. Thus, we resuspended viruses with medium after using the Dynabeads Intact Virus Enrichment kit. This Dynabeads treatment reduced soluble dsDNA in media of VeroE6/TMPRSS2 cells incubated with SARS-CoV-2 (for three days), which functions as DAMPs (Supplementary Fig. S4A), and TUNEL-positive injured cells were not affected by Dynabeads treatment (Supplementary Fig. S4B). These findings indicate that SARS-CoV-2 itself might affect TLR3 and TLR4 signaling.

### TLR4 inhibitor and IL-1R antagonist protect SARS-CoV-2-induced renal cell injury via the regulation of STAT3/NF-kB signaling

**Fig. 5:**
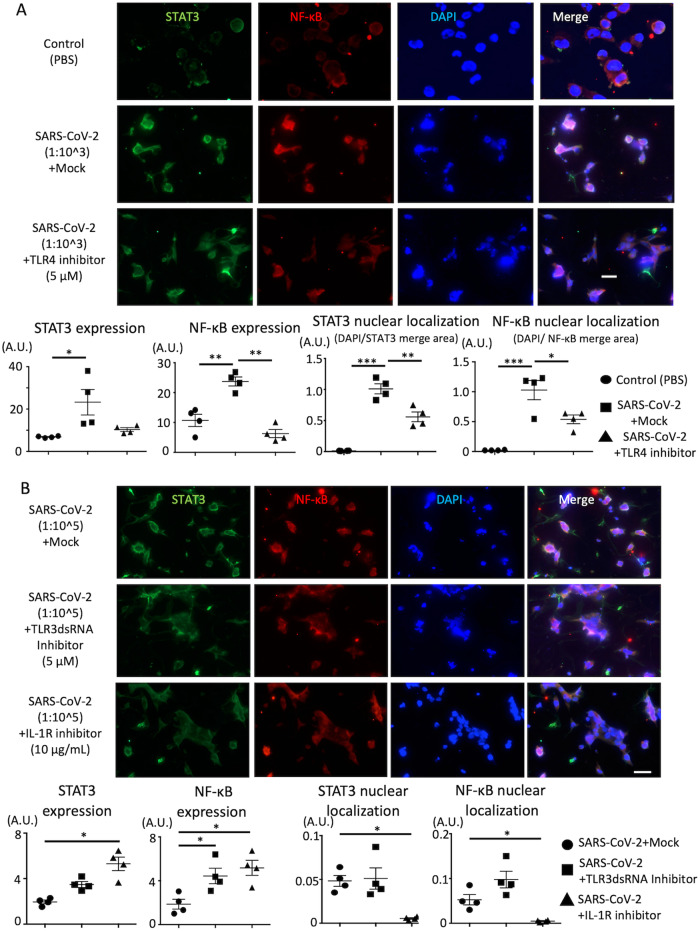
The expression of STAT3 and NF-κB in HK2 cells incubated with SARS-CoV-2. **A** The immunostaining of STAT3 and NF-κB in HK2 cells incubated with SARS-CoV-2 (dilution/1:10^3^) treated with mock or TLR4 inhibitor (for 72 h incubation). **B** The immunostaining of STAT3 and NF-κB in HK2 cells incubated with SARS-CoV-2 (dilution/1:10^5) treated with mock or TLR3dsRNA inhibitor or IL-1R antagonist (for 72 h incubation). The lower figures show the area of STAT3, NF-κB, STAT3-nuclear localization and NF-κB-nuclear localization. One-way ANOVA with post hoc Tukey’s test was performed for statistical analyses and significance was defined as *P < 0.05, **P < 0.01, ***P < 0.001. The scale bar is 50 μm.

Based on drug screening, we found that renal cell injury in response to SARS-CoV-2 develops via TLR and IL-1R. Mechanistic network analysis displayed that anakinra (IL-1R antagonist) and resatorvid (TLR4 inhibitor) are involved in NF-kB signaling as a common pathway (Fig. 3F, G). We examined the activation of NF-kB and signal transducer and activator of transcription 3 (STAT3) in cells incubated with SARS-CoV-2. Immunostaining revealed that the expression of NF-kB and STAT3 was increased, and nuclear translocation of these proteins was observed in HK2 cells incubated with SARS-CoV-2 (Fig. 5A). The TLR4 inhibitor suppressed the enhanced expression and nuclear translocation of NF-kB and STAT3 in HK2 cells incubated with a high dose of SARS-CoV-2 (Fig. 5A). IL-1R antagonist inhibited nuclear translocation but did not affect the increased expression of NF-kB and STAT3 in HK2 cells incubated with a low dose of SARS-CoV-2 (Fig. 5B). However, the TLR3dsRNA inhibitor did not affect NF-kB and STAT3 signaling (Fig. 5B). In addition, we tested the involvement of NF-kB and STAT3 signaling in HUEhT cells incubated with SARS-CoV-2 and found that only IL-1R antagonist regulated the nuclear translocation of NF-kB and STAT3 (Supplementary Fig. S5).

## Discussion

COVID-19 complicated with AKI contributes to increased mortality of affected patients. Various factors, including viral attack, excessive immune responses, drugs, and coagulopathy, may be involved in the pathogenesis of AKI [2]. Importantly, viral RNA and spike protein deposits are detected in kidneys from COVID-19 autopsies [16], implying that SARS-CoV-2 directly infects renal cells via the expression of ACE2 and CD147, resulting in the development of AKI [8]. However, these mechanisms have mainly been hypothesized by indirect evidence based on human histological analysis, and the precise molecular mechanisms remain poorly understood. Hence, we showed that in vitro SARS-CoV-2 predominantly injures tubular epithelial cells rather than the endothelium independently of viral replication in these cell lines. IPA revealed the involvement of TLR4/3 and IL-1R signaling in SARS-CoV-2-induced tubular injury, and these inhibitors protected against renal cell injury via the regulation of activated NF-kB and STAT3 pathways. In particular, TLR4 and TLR3 inhibition protects injured tubular epithelial cells, and IL-1R antagonist ameliorates tubular cell and endothelial cell injury. Mechanistically, TLR3 directly recognizes viral ssRNA [17] and induces host cell death and inflammatory responses [18, 19], whereas TLR4 and IL-1R sense DAMPs and IL-1, respectively, activating NF-kB signaling. SARS-CoV-2 accesses cells via entry molecules, including ACE2, and infiltrates via proteolytic virus activation by host proteases, including TMPRSS2, which cleaves the S protein, resulting in viral infection and replication [20]. The web database (https://www.proteinatlas.org/) revealed that renal cells, including tubular epithelial and endothelial cells, highly express these receptors. However, the expression levels of these proteases in renal cells are relatively low compared to those in the lung epithelial cells. Considering our data and renal cell characteristics, it is supposed that during SARS-CoV-2 viremia, the virus directly binds to the endothelium and tubular epithelium in the kidney via entry molecules. TLR3 recognizes viral RNA, leading to cell necrosis and the release of DAMPs [21]. TLR4 recognizes necrotic cell-derived DAMPs, including HMGB1, initiating inflammation via the activation of NF-kB. A recent study indicates that SARS-CoV-2 binds to and activates TLR4 via its spike glycoprotein to increase the expression of ACE2 [22]. Moreover, TLR3- and TLR4-mediated NF-kB activation promotes the generation of TNF-α and IL-1α, which stimulate TNFR and IL-1R as autocrine agents, resulting in further inflammation and cell necrosis [23] (Supplementary Fig. S6). However, no SARS-CoV-2 replication in renal cells in our study conflicts with a recent report that human-induced pluripotent-stem-cell (iPSC)-derived kidney organoids were infected with SARS-CoV-2 [9]. The discrepancy is likely due to the differences in cell condition; the immortalized cells in our studies might be resistant to viral infection, and conversely, iPSC cells might be susceptible to the virus. It is known that iPSC-derived kidney organoids are mainly composed of fetal kidney cells, which have different biological functions and characteristics to native renal cells [24], which might affect the response to virus. Thus, these results should be carefully interpreted. Meanwhile, our data showed that tubular epithelia are more sensitive to SARS-CoV-2 than the endothelia. This is compatible with human data, in which the deposition of SARS-CoV-2 is frequently detected in the renal tubules [25]. Furthermore, numerous inflammatory cells infiltrate necrotic tubular cells. This coincides with our RNA-seq data that revealed that the expression levels of proinflammatory genes were upregulated in tubular cells incubated with SARS-CoV-2, indicating that SARS-CoV-2 passes through the impaired glomerular filtration barrier [26] (due to multi-factor, including sepsis, cytokines, activated immune cells, and thrombotic events, rather than viral direct attack), reaches the tubules, and induces tubular injury. We first demonstrated that TLR and IL-1R signaling and downstream pathways in renal cell may be novel therapeutic targets in SARS-CoV-2 mediated kidney injury, although these pathways have been reportedly associated with lung epithelial injury [27, 28]. In particular, the production of interleukin-1β (IL-1β) in coronavirus infection is involved in the exacerbation of pulmonary injury and leads to respiratory failure and widespread thrombosis [27]. In addition, experimental evidence demonstrated that the viral spike protein of SARS-CoV-2 binds with TLRs including TLR4, and the interaction could result in a cytokine storm. Thus, these might be a suitable target for regulating severe COVID-19 [29]. Moreover, TLR4-knockout mice exhibited improved mortality compared to wild-type mice in response to SARS-CoV-1, which is similar to SARS-CoV-2 [28]. Meanwhile, our data demonstrated that SARS-CoV-2-mediated injured cells were not influenced by Dynabeads treatment that reduced soluble dsDNA, indicating that SARS-CoV-2 directly initiated renal cell necrosis and caused further cellular injury via DAMPs (dsDNA)-mediated TLR activation. However, because its treatment could not entirely remove all DAMPs, SARS-CoV-2 solution containing some DAMPs might affect renal cells via TLR4 [28].

A clinical observational study that IL-1R antagonists reduced the requirement of mechanical ventilation and decreased mortality in patients with COVID-19 [30] supports our data. In addition, autopsy findings displayed that an activated STAT3 pathway was involved in the kidney of patients with COVID-19 [31], which is compatible with our study. ACE2-humanized mice develop renal tubular injury after SARS-CoV-2 infection [32], thus in vivo studies are required to evaluate the efficacy of these targets in the future. The incidence of AKI in patients with COVID-19 is relatively high (3–34%). COVID-19 AKI is associated with increased mortality rates and is an independent risk factor for all-cause mortality [33]. Globally, researchers focus on the development of antiviral drugs or vaccines to prevent viral infection [34]. However, regardless of these approaches, some patients are resistant to these drugs, or delays in diagnosis may affect their response to antiviral medications, resulting in multi-organ failure, leading to severe illness. Thus, organ-specific therapies for diseases, including AKI, are needed to improve the patient outcomes. Through comprehensive analysis and in vitro experiments, we discovered novel therapeutic targets against SARS-CoV-2-mediated kidney injury. Our results indicate that the inhibition of TLR and IL-1R signaling pathways may contribute to the improvement of SARS-CoV-2-mediated kidney injury.

## Material and methods

### Cell lines and co-culture of renal cells with SARS-CoV-2 solution

HK2 (human) tubular cells (1 × 10^5^ cells/mL) were grown in an epithelial basal medium (Thermo Fisher Scientific, Waltham, MA, USA) as described previously [35]. HUEhT cells (Japanese Collection of Research Bioresources Cell Bank) were cultured in medium (see Supplementary Materials) at 1 × 10^5^ cells/mL concentration as described [36]. As SARS-CoV-2 infects VeroE6/TMPRSS2 cell which was obtained from the JCRB (Osaka, Japan; Cell number: JCRB1819), this cell line was used as a positive control cell. These cells were seeded into culture plates until they reached approximately 80–90% confluence. Each cell line was cultured with various diluted SARS-CoV-2 solutions for 24–72 h at 37 °C. To prepare the SARS-CoV-2-containing medium, SARS-CoV-2 (strain: JPN/TY/WK-521) was given by the NIID (Tokyo, Japan). SARS-CoV-2 was inoculated into VeroE6/TMPRSS2 cells cultured in virus growth medium of which composition was described previously [37].

### The dilution of SARS-CoV-2-containing medium and the evaluation of CPE in renal cells incubated with SARS-CoV-2

The cell culture supernatant of SARS-CoV-2-inoculated VeroE6/TMPRSS2 cells was collected as the SARS-CoV-2 solution after incubation. Its viral titer was determined to be 7 log_10_ TCID_50_/mL on the basis of CPE seen in VeroE6/TMPRSS2 cells. For experiments using HK2 and HUEhT cells, the SARS-CoV-2 solution (7 log_10_ TCID_50_/mL) was diluted in Dulbecco’s modified Eagle’s medium (DMEM; Nissui Pharmaceutical, Tokyo, Japan) from 10^−1^ to 10^−7^.

### Microscopic analysis of renal cells incubated with SARS-CoV-2 solution and pretreatment of Dynabeads Intact Virus Enrichment kit

To perform microscopic analysis, HK2 and HUEhT cells were seeded in a 96-well microplate or 8-channel culture slide (1 × 10^5^ cells/mL) and co-cultured with SARS-CoV-2 solution. Phase-contrast images in microscopy were taken to assess the morphology in the cells. To investigate the effects of DAMPs in the SARS-CoV-2-containing medium, the medium was treated with Dynabeads Intact Virus Enrichment kit (optimized for SARS-CoV-2) (Thermo Fisher Scientific). The enriched SARS-CoV-2 was then resuspended in DAMPs-free medium.

### Measurement of dsDNA, TUNEL assay, and Immunostaining

Soluble dsDNAs in the supernatant were evaluated by dsDNA Assay kit (see Supplementary Materials) [35]. Cell injury and the associated signal were evaluated by TUNEL assay [35] and immunostaining of NF-κB p65 subunit and STAT3 using anti-NFκB antibody (Abcam, Cambridge, UK) and anti-STAT3 antibody (Thermo Fisher Scientific), respectively, after fixation with 4% paraformaldehyde. The positive areas of TUNEL staining and immunostaining were measured using the ImageJ software as described [38]. The data were expressed as the mean ± standard error of the mean (SEM) from three independent experiments.

### Evaluation of infectious and proliferative capacities of SARS-CoV-2 within HK2 cells, HUEhT cells and VeroE6/TMPRSS2 cells

HK2, HUEhT, and VeroE6/TMPRSS2 cells were exposed to a diluted solution of SARS-CoV-2 (1 ×10^−1^). One hour after exposure, the SARS-CoV-2 solution was removed and replaced with virus-free DMEM. After 0-, 23- and 71-h cell incubation, the supernatant was then removed and total RNA was extracted from each cell using the RNA isolation regent (see Supplementary Materials) as previously described [38]. First-strand cDNA was synthesized using cDNA Synthesis kit (see Supplementary Materials), and quantitative PCR was performed using qPCR kit (see Supplementary Materials). The relative amounts of viral genome and its transcripts were normalized based on the transcript level of 18s rRNA (primers and probe kit: EUK 18S rRNA OLIGO MIX, Thermo Fisher Scientific). The information of primers and probe targeting the SARS-CoV-2 N gene was in Supplementary Materials [39]. To confirm whether infectious SARS-CoV-2 could be recovered from each virus-inoculated cell, each cells were cultured in SARS-CoV-2-free medium for 72 h after 1-h co-culture with SARS-CoV-2. The culture supernatants or lysates of these cells were inoculated into VeroE6/TMPRSS2 cells. These cell lysates were prepared by freezing and thawing of cells resuspended in DMEM three times, and the supernatants were also frozen and thawed three times. After 72 h of incubation, the presence or absence of CPE on VeroE6/TMPRSS2 cells incubated with each cell’s supernatant or lysate was evaluated.

Furthermore, VeroE6/TMPRSS2, HK2, or HUEhT cells were co-cultured with SARS-CoV-2 solution for 1 or 24 h. After removing the SARS-CoV-2 solution and washing twice with phosphate-buffered saline, the SARS-CoV-2 N protein in each cell was labeled using antibodies for immunostaining (see Supplementary Materials).

### RNA isolation and RNA sequencing of HK2 and HUEhT cell

To analyze the transcriptome, HK2 cells (3 × 10^5^ cells/well) and HUEhT cells (2.2 × 10^5^ cells/well) were incubated with diluted SARS-CoV-2 solution (10^−3^) for 72 h. Total mRNA was extracted using the ISOGEN II Kit. The RNA quality was evaluated by RNA integrity number (RIN) and library preparation was performed (see Supplementary Materials) as previously described [38]. The libraries were sequenced on sequencing system (see Supplementary Materials) in 2 × 150 bp setting. The following data analysis were performed as previously described [38]. In short, the sequenced reads were trimmed using cutadapt [40] and mapped to human reference genome (hg38) using STAR aligner [41]. DEGs were identified by DESeq2 library [42]. |log_2_ fold-change (FC)| ≥ 1 and adjusted *p*-value < 0.05 were used as a cut-off value for finding DEGs. GO analysis was conducted on DEGs using gprofiler2 [43]. The sequencing data can be downloaded from (Accession Number GSE202095).

### Ingenuity pathway analysis (IPA)

Upstream pathway and canonical pathway analyses of DEGs were conducted using analysis system (Supplementary Materials), as previously described [38]. DEGs with |log_2_FC| ≥ 1.5 and a false discovery rate ≤ 0.05 was used as the inputs.

### Evaluation of potential therapeutic targets against SARS-CoV-2-induced renal cell injury

The cells were pre-incubated with anakinra (an IL-1R inhibitor; MedChemExpress, South Brunswick, NJ, USA), TLR3/dsRNA complex inhibitor (Merck Millipore, Germany), TAK-242 (a TLR4 inhibitor; Merck Millipore, Germany), and infliximab (an anti-TNF-α monoclonal antibody; MedChemExpress) for 30 min. The cells were then treated with SARS-CoV-2 solutions at various dilution ratios and cultured for 48 or 72 h in the presence of each inhibitor. The cytotoxicity of the inhibitors was assessed using the Cell Counting Kit8 in a concentration-dependent manner, and the optimal dosage was determined.

### Flow cytometric analysis of TLR3 in renal cells

The expression of surface TLR3 in HK2 and HUEhT cells was analyzed using flow cytometer (used antibodies; see Supplementary Materials). Adherent cells were treated with accutase to detach them, and then the detached cells were incubated with the antibodies.

### Statistical analyses

Statistical analyses were conducted using computer system (see Supplementary Materials). To determine statistical significance, one-way analysis of variance with Tukey’s posthoc or Dunnett’s multiple comparisons test, or t-test was performed with a significance threshold set at *p* < 0.05. The *p*-values were adjusted for multiple hypothesis testing using the Benjamini–Hochberg method. All data were presented as mean ± SEM.

### Supplementary information

Supplementary materials

Supplemental figure and table legend

Supplemental table1,2

Supplementary Figure S1

Supplementary Figure S2

Supplementary Figure S3

Supplementary Figure S4

Supplementary Figure S5

Supplementary Figure S6

## Data Availability

The data supporting the findings of this study are available from the corresponding author upon reasonable request.
