# Supplementary material for: Inhibition of Toll-like receptor 4 and Interleukin-1 receptor prevent SARS-CoV-2 mediated kidney injury
Source: Cell Death Discov. 2023 Aug 10;9:293. doi: 10.1038/s41420-023-01584-x (PMC10415265; doi:10.1038/s41420-023-01584-x)
Supplement: Supplementary file 1 — Supplementary materials [file 41420_2023_1584_MOESM1_ESM.docx]

Supplementary Materials

HUEhT cells medium; HuMedia-EG2 (Kurabo, Osaka, Japan)

dsDNA Assay kit; the PICO Green dsDNA Assay Kit (Thermo Fisher Scientific)

RNA isolation regent; ISOGEN II kit (Nippon Gene, Tokyo, Japan),

cDNA Synthesis kit; the FastGene cDNA Synthesis 5× ReadyMix OdT (NIPPON Genetics, Tokyo, Japan)

qPCR kit; FastGene™ QPCR Probe Mastermix w/ROX (NIPPON Genetics).

The primers and probe targeting the SARS-CoV-2 N gene

Forward primer; NIID_2019-nCoV_N_F2 [AAATTTTGGGGACCAGGAAC]

Reverse primer; NIID_2019-nCoV_N_R2 [TGGCAGCTGTGTAGGTCAAC]

Probe; NIID_2019-nCoV_N_P2 [FAM-ATGTCGCGCATTGGCATGGA-TAMRA]

Antibodies for immunostaining of SARS-CoV-2 N gene

Primary antibody; SARS-CoV-2 (2019-nCoV) Nucleoprotein/NP Antibody, Rabbit Mab (Sino Biological, Beijing, China)

Secondary antibody; Anti-Rabbit IgG FITC conjugate (Sigma-Aldrich, St. Louis, MO, USA).

RNA library preparation; NEBNext Ultra II Directional RNA Library Prep Kit for Illumina (New England Biolabs; #E7760S/L)

Sequencing system; NovaSeq 6000 platform

Upstream pathway and canonical pathway analyses of DEGs; Ingenuity IPA (QIAGEN Inc., Redwood City, CA)

Antibody for TLR3 in flow cytometer; PE-labeled anti-human TLR3 or isotype control antibodies (BioLegend, San Diego, CA, USA)

Computer system of statistical analyses; R v3.6.1 (R Foundation for Statistical Computing, Vienna, Austria; https://www.R-project.org/) or Prism 9 (GraphPad Software, San Diego, CA, USA).
