# Supplementary material for: Inhibition of Toll-like receptor 4 and Interleukin-1 receptor prevent SARS-CoV-2 mediated kidney injury
Source: Cell Death Discov. 2023 Aug 10;9:293. doi: 10.1038/s41420-023-01584-x (PMC10415265; doi:10.1038/s41420-023-01584-x)
Supplement: Supplementary file 2 — Supplemental figure and table legend [file 41420_2023_1584_MOESM2_ESM.docx]

**Inhibition of Toll-like receptor 4 and Interleukin-1 receptor prevent SARS-CoV-2 mediated kidney injury**

**Supplementary Figure Legend**

**Supplementary Figure S1.** **A. Gene expression levels (FPKM values) of HK2 and HUEhT cells incubated with Mock or SARS-CoV-2 in RNA-sequence. B. The cytotoxicity of IL-1Rantagonist, TNF-α inhibitor, TLR4 inhibitor, and TLR3 inhibitor in HK2 and HUEhT cells in a dose dependent manner (for 48 and 72 h) by Cell Counting Kit8**

FPKM; fragments per kilobase of exon per million reads mapped

**Supplementary Figure S2**. **The drug efficacy (IL-1R antagonist, TNF-α inhibitor, TLR4 inhibitor, and TLR3 inhibitor) in HK2 and HUEhT cells incubated with SARS-CoV-2 (for 48 and 72 h).** Cell morphology was assessed by light microscopy. A. HK2 cell. B. HUEhT cell. +: abnormal cell morphology; ±: uncertain abnormal cell morphology; -: no abnormal cell morphology. C. Flow cytometric analysis of TLR3 expression in HK2 and HUEhT cell. T test was performed for statistical analyses, and significance was defined as ***P* < 0.01.

**Supplementary Figure S3.** **TUNEL staining in HUEhT cells incubated with SARS-CoV-2 treated with inhibitor**

HUEhT cells incubated with SARS-CoV-2 (dilution/1:10^2) treated with mock or TLR4 inhibitor or TLR3dsRNA inhibitor or IL-1R antagonist (for 72 h incubation). Lower graphs show TUNEL positive area TUNEL/Green, DAPI/Blue. One-way ANOVA with post hoc Dunnett's multiple comparisons test was performed for statistical analyses, and significance was defined as **P* < 0.05. ***P* < 0.01. NS: Not significant. The scale bar is 200 μm.

**Supplementary Figure S4. TUNEL staining under phase-contrast microscopy of HK2 cells treated with optimized for SARS-CoV-2-solution**

A. Soluble dsDNA in media of VeroE6/TMPRSS2 cells. From left, media of VeroE6/TMPRSS2 cells incubated with mock (for 3 days), media of VeroE6/TMPRSS2 cells incubated with SARS-CoV-2 (for 3 days), and media of VeroE6/TMPRSS2 cells incubated with SARS-CoV-2 (for 3 days) treated with Dynabeads Intact Virus Enrichment kit. B. Figures and graph show TUNEL staining and area of HK2 cells treated with SARS-CoV-2 solution with or without pretreatment of Dynabeads. Upper figures and graph; SARS-CoV-2 solution (1:10^5), Lowe figures and graph; SARS-CoV-2 solution (1:10^3). T test was performed for statistical analyses. NS: Not significant. The scale bar is 100 μm.

**Supplementary Figure S5.** **The expression of STAT3 and NF-κB in HUEhT cells incubated with SARS-CoV-2.** The immunostaining of STAT3 and NF-κB in SARS-CoV-2 (dilution/1:10^2)-treated HUEhT cells with mock or TLR4 inhibitor or TLR3dsRNA inhibitor or IL-1R antagonist (for 72 h incubation). Lower figures show the area of STAT3, NF-κB, STAT3-nuclear localization and NF-κB -nuclear localization. One-way ANOVA with post hoc Tukey’s test was performed for statistical analyses, and significance was defined as **P* < 0.05, ***P* < 0.01. The scale bar is 50 μm.

**Supplementary Figure S6.** **Schematic mechanism of SARS-CoV-2-mediated tubular epithelial cell injury.** SARS-CoV-2 binds to receptor (including ACE2, CD147 and Kim1) and injures epithelial cells without virus replication. TLR3 recognize virus ssRNA, which initiates cell death and activates NF-κB pathway. DAMPs including HMGB1 released from injured cells bind to TLR4 and activate NF-κB pathway. Upregulated IL1 and TNF mediated by NF-κB activation binds to receptors in an autocrine signaling, causing pro-inflammatory response via the activated STAT3-NF-κB pathway. TLR3dsRNA inhibitor, TLR4 inhibitor and IL-1R antagonist regulates these signaling pathway.

Abbreviation; ACE2; angiotensin converting enzyme 2, Kim1; kidney injury molecule 1, TLR; Toll-like receptor, NF-κB; Nuclear factor kappa B, DAMPs; Damage-associated molecular patterns, HMGB1; High mobility group box 1, IL-1; Interleukin 1, TNF; tumor necrosis factor, STAT3; signal transducer and activator of transcription 3.

**Supplementary materials**

Supplementary Table S1A. Upstream regulator analysis in HK2 cells incubated with compared to HK2 cells incubated with Mock (Top50).

Supplementary Table S1B. Causal Network Analysis in HK2 cells incubated with compared to HK2 cells incubated with Mock (Top50).
