# Supplementary material for: Inhibition of Toll-like receptor 4 and Interleukin-1 receptor prevent SARS-CoV-2 mediated kidney injury
Source: Cell Death Discov. 2023 Aug 10;9:293. doi: 10.1038/s41420-023-01584-x (PMC10415265; doi:10.1038/s41420-023-01584-x)
Supplement: Supplementary file 3 — Supplemental table1,2 [file 41420_2023_1584_MOESM3_ESM.docx]

Supplementary data

**Supplemental Table S1A. Upstream regulator analysis (Top 50)**

| Upstream Regulator | Expr Log Ratio | Predicted Activation State | Activation z-score | Flags | p-value of overlap | Mechanistic Network |
| --- | --- | --- | --- | --- | --- | --- |
| lipopolysaccharide |  | Inhibited | -8.135 |  | 1.1E-24 | 216 (14) |
| IL1B |  | Inhibited | -6.919 |  | 3.66E-30 | 211 (13) |
| TNF | -2.672 | Inhibited | -6.333 |  | 1.54E-34 | 207 (12) |
| poly rI:rC-RNA |  | Inhibited | -6.072 | bias | 1.22E-15 | 213 (13) |
| NFkB (complex) |  | Inhibited | -5.989 | bias | 4.25E-24 | 204 (15) |
| IL1A |  | Inhibited | -5.381 | bias | 2.66E-26 | 207 (13) |
| tetradecanoylphorbol acetate |  | Inhibited | -5.235 |  | 7.02E-15 | 209 (13) |
| IFNG |  | Inhibited | -4.995 |  | 8.14E-27 | 209 (12) |
| E. coli B5 lipopolysaccharide |  | Inhibited | -4.902 |  | 3.07E-11 | 193 (13) |
| IL1 |  | Inhibited | -4.889 |  | 6.86E-20 | 190 (12) |
| STAT3 | -0.34 | Inhibited | -4.816 |  | 8.65E-12 | 224 (15) |
| PDGF BB |  | Inhibited | -4.812 |  | 1.02E-14 | 205 (16) |
| CEBPB | -1.049 | Inhibited | -4.602 | bias | 1.39E-11 | 251 (17) |
| RELA | -0.309 | Inhibited | -4.581 | bias | 3.52E-16 | 193 (13) |
| F2 |  | Inhibited | -4.569 | bias | 1.54E-17 | 147 (15) |
| 8-bromo-cAMP |  | Inhibited | -4.45 | bias | 6.99E-09 | 293 (23) |
| Salmonella enterica serotype abortus equi lipopolysaccharide |  | Inhibited | -4.42 | bias | 3.7E-17 | 200 (16) |
| IL6 | -4.461 | Inhibited | -4.359 |  | 1.27E-15 | 166 (16) |
| MYD88 | 0.262 | Inhibited | -4.32 | bias | 2.36E-12 | 195 (14) |
| IL17A |  | Inhibited | -4.301 |  | 3.36E-14 | 196 (12) |
| TLR7 |  | Inhibited | -4.236 | bias | 4.08E-08 | 151 (13) |
| OSM |  | Inhibited | -4.176 |  | 1.58E-10 | 156 (16) |
| TGFB1 | -0.139 | Inhibited | -4.152 |  | 2.88E-19 | 279 (17) |
| TLR4 |  | Inhibited | -4.152 | bias | 1.16E-13 | 220 (14) |
| PGR |  | Inhibited | -4.139 |  | 1.07E-09 | 279 (20) |
| beta-estradiol |  | Inhibited | -4.129 |  | 6.41E-23 | 277 (17) |
| CG |  | Inhibited | -4.102 |  | 2.54E-18 | 241 (15) |
| EGF |  | Inhibited | -4.043 |  | 7.19E-11 | 217 (19) |
| E. coli serotype 0127B8 lipopolysaccharide |  | Inhibited | -3.972 |  | 9.56E-09 | 176 (12) |
| P38 MAPK |  | Inhibited | -3.963 | bias | 2.2E-12 | 163 (13) |
| resiquimod |  | Inhibited | -3.937 |  | 8.41E-17 | 192 (14) |
| TLR3 | -0.711 | Inhibited | -3.936 | bias | 1.64E-08 | 174 (13) |
| TLR9 |  | Inhibited | -3.891 | bias | 0.00000032 | 160 (17) |
| CHUK | 0.034 | Inhibited | -3.851 | bias | 6.29E-12 | 198 (14) |
| Jnk |  | Inhibited | -3.816 |  | 7.92E-09 | 219 (20) |
| KITLG | 1.642 | Inhibited | -3.797 | bias | 0.00000289 | 172 (19) |
| STAT1 | -0.561 | Inhibited | -3.783 |  | 9.32E-07 | 197 (13) |
| Tlr |  | Inhibited | -3.767 | bias | 4.36E-08 | 164 (17) |
| F2R | -0.766 | Inhibited | -3.748 |  | 1.16E-11 | 144 (16) |
| forskolin |  | Inhibited | -3.725 |  | 1.36E-13 | 212 (21) |
| IL15 | 0.376 | Inhibited | -3.714 |  | 2.75E-09 | 192 (15) |
| STAT4 | -0.275 | Inhibited | -3.681 | bias | 0.0000609 | 185 (16) |
| IKBKG | 0.695 | Inhibited | -3.67 | bias | 6.79E-12 | 228 (15) |
| salmonella minnesota R595 lipopolysaccharides |  | Inhibited | -3.657 | bias | 2.73E-11 | 140 (18) |
| CD40LG |  | Inhibited | -3.648 | bias | 1.18E-11 | 213 (15) |
| ZFTA-RELA |  | Inhibited | -3.606 | bias | 9.74E-11 |  |
| NFAT5 | -0.441 | Inhibited | -3.6 |  | 6.25E-14 | 159 (17) |
| 2-(4-amino-1-isopropyl-1H-pyrazolo[3,4-d]pyrimidin-3-yl)-1H-indol-5-ol |  | Inhibited | -3.569 |  | 0.000773 | 124 (7) |
| IL33 |  | Inhibited | -3.536 |  | 4.88E-15 | 165 (15) |
| TLR2 | 0.959 | Inhibited | -3.502 |  | 4.79E-09 | 144 (13) |

**Supplemental Table S1B. Upstream regulator by Causal Network Analysis (Top 50)**

| Master Regulator | Depth | Predicted Activation | Activation z-score | p-value of overlap | Network bias-corrected p-value | Causal network | Target-connected regulators |
| --- | --- | --- | --- | --- | --- | --- | --- |
| 2M-TNF | 3 | Activated | 5.661 | 8.41E-38 | 1.00E-04 | 185 (15) | 14 |
| infliximab | 2 | Activated | 5.895 | 1.90E-32 | 1.00E-04 | 137 (4) | 4 |
| tetrandrine | 2 | Activated | 6.561 | 3.50E-32 | 1.00E-04 | 145 (10) | 10 |
| adalimumab | 2 | Activated | 5.874 | 6.78E-32 | 1.00E-04 | 134 (2) | 2 |
| etalocib | 3 | Activated | 5.439 | 8.33E-31 | 1.00E-04 | 106 (12) | 11 |
| anakinra | 2 | Activated | 7.637 | 9.17E-30 | 1.00E-04 | 107 (5) | 5 |
| COMMD1 | 2 | Activated | 6.807 | 7.94E-29 | 1.00E-04 | 94 (5) | 5 |
| SKLB023 | 2 | Activated | 6.765 | 2.10E-28 | 1.00E-04 | 84 (5) | 5 |
| SIGLEC7 | 3 | Activated | 4.459 | 3.23E-27 | 1.00E-04 | 136 (35) | 33 |
| TWF2 | 3 | Activated | 5.222 | 3.69E-27 | 1.00E-04 | 81 (11) | 10 |
| Vhl | 2 | Activated | 6.399 | 3.82E-27 | 1.00E-04 | 85 (6) | 6 |
| glycyrrhizic acid | 2 | Activated | 6.54 | 6.52E-27 | 1.00E-04 | 87 (9) | 9 |
| RCAN1 | 2 | Activated | 6.464 | 8.30E-27 | 1.00E-04 | 95 (11) | 11 |
| FOXD1 | 2 | Activated | 6.022 | 1.61E-26 | 1.00E-04 | 139 (6) | 5 |
| FOXJ1 | 2 | Activated | 5.853 | 2.23E-26 | 1.00E-04 | 139 (7) | 7 |
| KSR2 | 2 | Activated | 6.582 | 2.89E-26 | 1.00E-04 | 75 (4) | 4 |
| ERBIN | 2 | Activated | 5 | 7.24E-26 | 1.00E-04 | 81 (6) | 6 |
| FOXP3 | 2 | Activated | 6.464 | 1.39E-25 | 1.00E-04 | 92 (11) | 11 |
| epicatechin | 2 | Activated | 6.399 | 1.57E-25 | 2.00E-04 | 85 (7) | 7 |
| ATG16L1 | 2 | Activated | 4.778 | 1.74E-25 | 1.00E-04 | 81 (6) | 6 |
| kenpaullone | 2 | Activated | 6.983 | 4.50E-25 | 1.00E-04 | 84 (6) | 5 |
| Gli | 2 | Activated | 3.395 | 8.23E-25 | 1.00E-04 | 68 (6) | 6 |
| bisindolylmaleimide II | 2 | Activated | 6.037 | 1.10E-24 | 1.00E-04 | 83 (3) | 2 |
| myricetin | 2 | Activated | 5.57 | 1.75E-24 | 2.00E-04 | 94 (12) | 12 |
| pepstatin | 2 | Activated | 6.045 | 2.10E-24 | 1.00E-04 | 74 (9) | 9 |
| milrinone | 2 | Activated | 6.29 | 2.54E-24 | 1.00E-04 | 71 (7) | 6 |
| CD200R1 | 2 | Activated | 6.957 | 2.55E-24 | 2.00E-04 | 90 (9) | 9 |
| ABI1 | 2 | Activated | 6.395 | 2.72E-24 | 1.00E-04 | 94 (6) | 5 |
| glaucocalyxin A | 2 | Activated | 6.454 | 3.16E-24 | 1.00E-04 | 70 (3) | 3 |
| KP-SD-1 | 2 | Activated | 5.584 | 3.52E-24 | 4.00E-04 | 97 (8) | 8 |
| resatorvid | 2 | Activated | 7.014 | 4.50E-24 | 8.00E-04 | 94 (10) | 10 |
| CRABP2 | 3 | Activated | 2.06 | 4.69E-24 | 4.00E-04 | 114 (17) | 17 |
| evodiamine | 2 | Activated | 4.778 | 5.31E-24 | 2.00E-04 | 81 (5) | 5 |
| delta-aminolevulinic acid | 2 | Activated | 6.814 | 5.82E-24 | 8.00E-04 | 91 (7) | 7 |
| PADI2 | 2 | Activated | 3.969 | 1.06E-23 | 1.00E-04 | 61 (2) | 2 |
| norisoboldine | 2 | Activated | 5.115 | 1.08E-23 | 1.00E-04 | 74 (6) | 6 |
| Alpha catenin | 2 | Activated | 6.814 | 1.52E-23 | 2.20E-03 | 91 (7) | 7 |
| ZIC1 | 2 | Activated | 2.75 | 1.54E-23 | 1.00E-04 | 64 (4) | 3 |
| procyanidin B2 | 2 | Activated | 5.598 | 1.68E-23 | 3.00E-04 | 83 (7) | 6 |
| chloramine | 2 | Activated | 5.742 | 1.70E-23 | 1.00E-04 | 67 (4) | 3 |
| HLX | 3 | Activated | 5.155 | 2.27E-23 | 6.00E-04 | 118 (8) | 8 |
| leptomycin B | 2 | Activated | 6.814 | 2.51E-23 | 1.00E-04 | 91 (8) | 8 |
| PD 168393 | 2 | Activated | 5.031 | 2.97E-23 | 2.00E-04 | 111 (14) | 14 |
| erlotinib | 2 | Activated | 3.669 | 3.40E-23 | 9.00E-04 | 113 (11) | 11 |
| TRIM37 | 2 | Activated | 6.018 | 3.52E-23 | 2.00E-04 | 61 (2) | 2 |
| sulfacetamide | 3 | Activated | 6.063 | 3.94E-23 | 2.00E-04 | 68 (9) | 6 |
| methazolamide | 3 | Activated | 6.063 | 3.94E-23 | 2.00E-04 | 68 (9) | 6 |
| xestospongin C | 2 | Activated | 6.254 | 4.26E-23 | 1.00E-04 | 89 (5) | 5 |
| magnesium sulfate | 2 | Activated | 5.25 | 4.28E-23 | 2.00E-04 | 61 (3) | 3 |
| thenoyltrifluoroacetone | 2 | Activated | 6.621 | 4.41E-23 | 2.00E-04 | 69 (3) | 3 |
