# Supplementary figures and images for: Inhibition of Toll-like receptor 4 and Interleukin-1 receptor prevent SARS-CoV-2 mediated kidney injury
Source: Cell Death Discov. 2023 Aug 10;9:293. doi: 10.1038/s41420-023-01584-x (PMC10415265; doi:10.1038/s41420-023-01584-x)

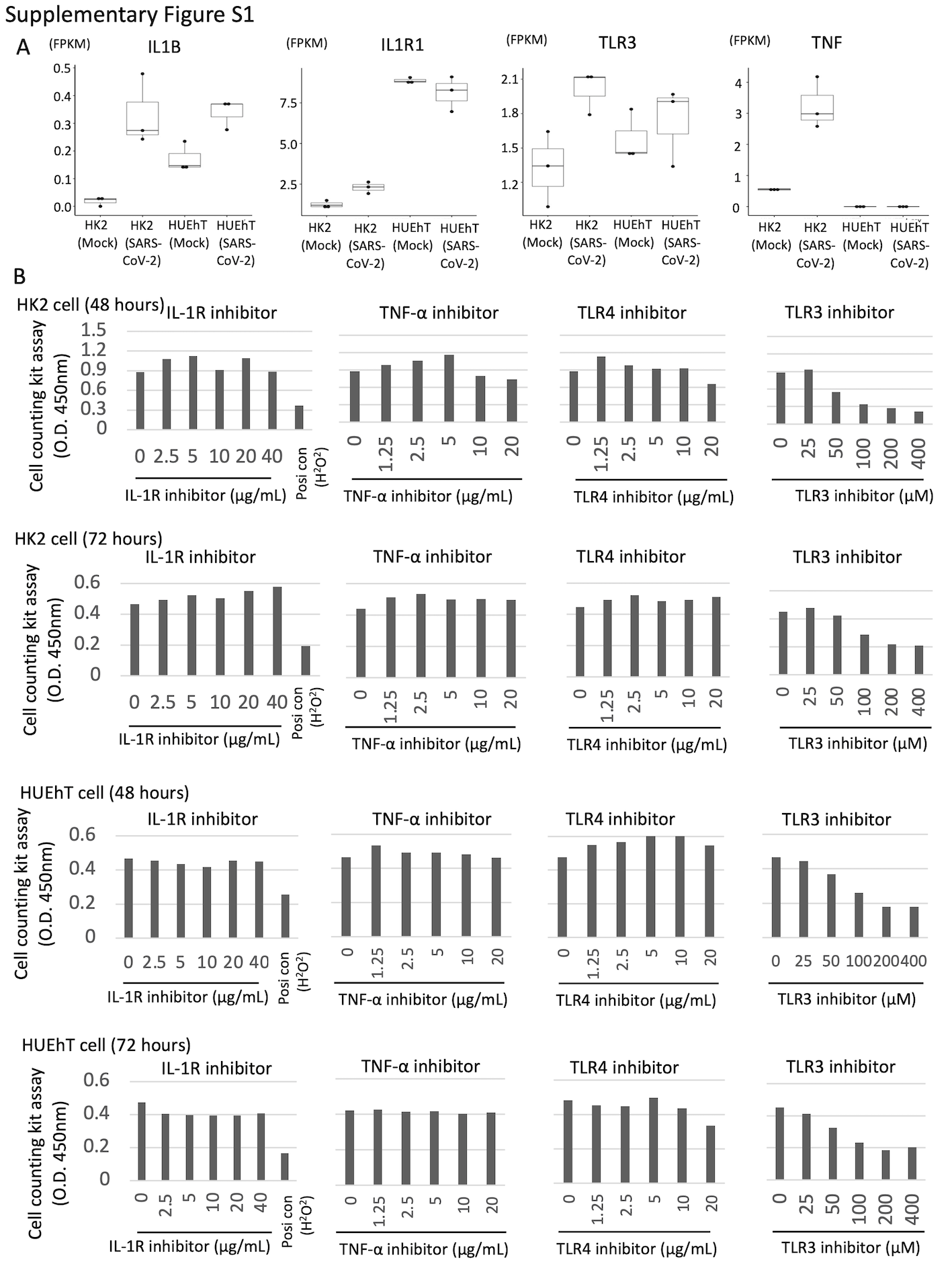

Supplement: Supplementary file 4 — Supplementary Figure S1 [file 41420_2023_1584_MOESM4_ESM.tif]

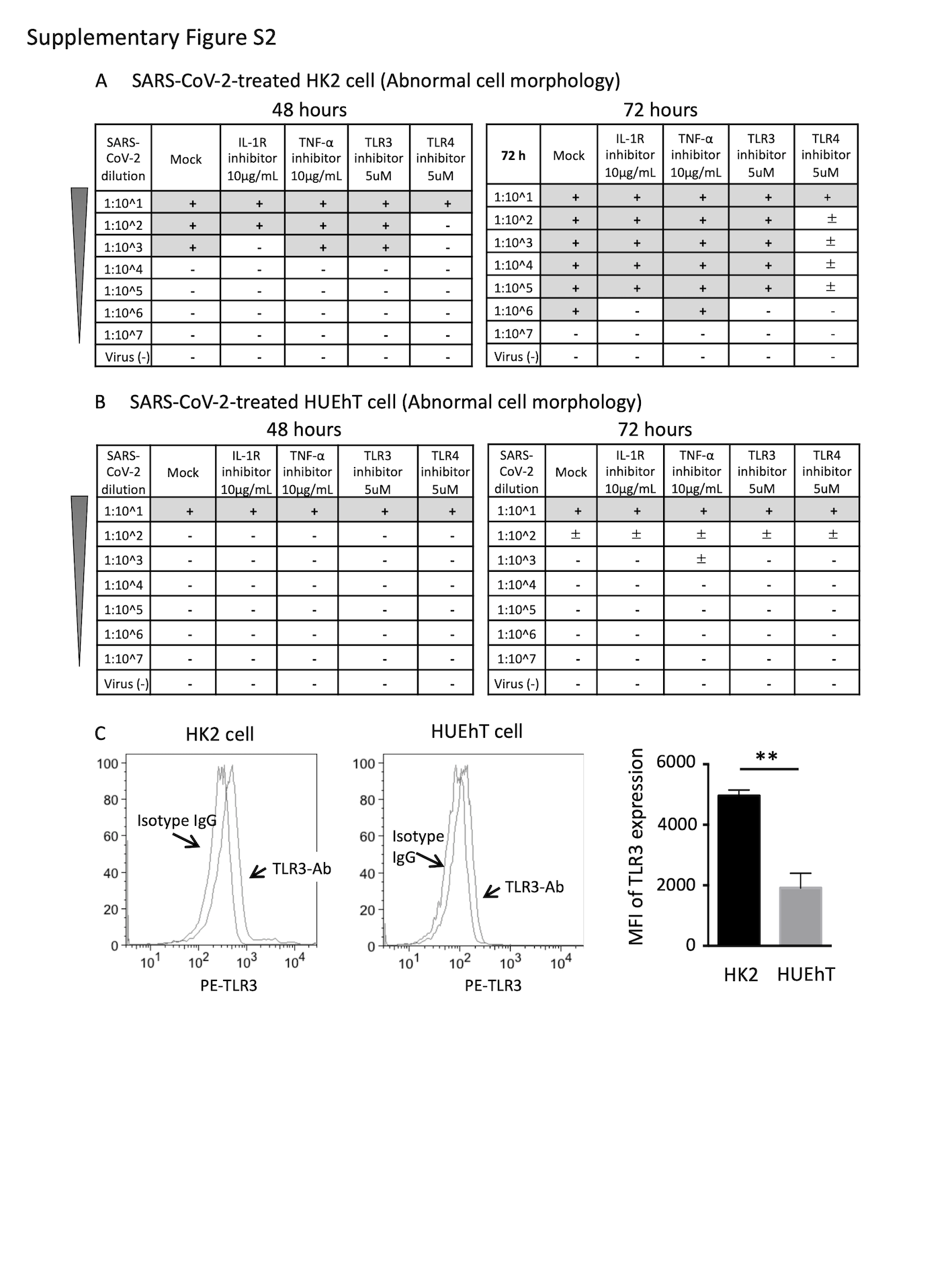

Supplement: Supplementary file 5 — Supplementary Figure S2 [file 41420_2023_1584_MOESM5_ESM.tif]

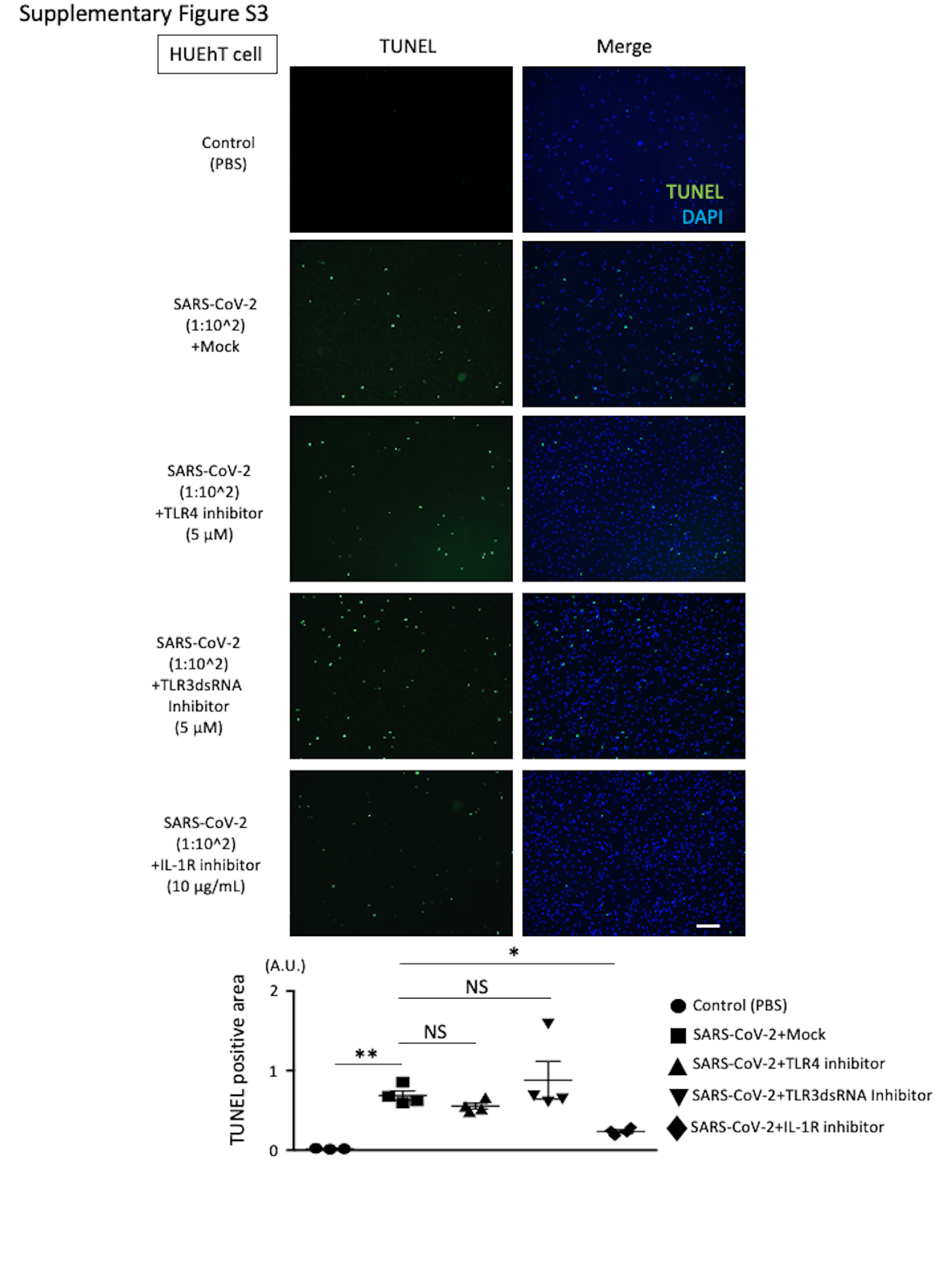

Supplement: Supplementary file 6 — Supplementary Figure S3 [file 41420_2023_1584_MOESM6_ESM.tif]

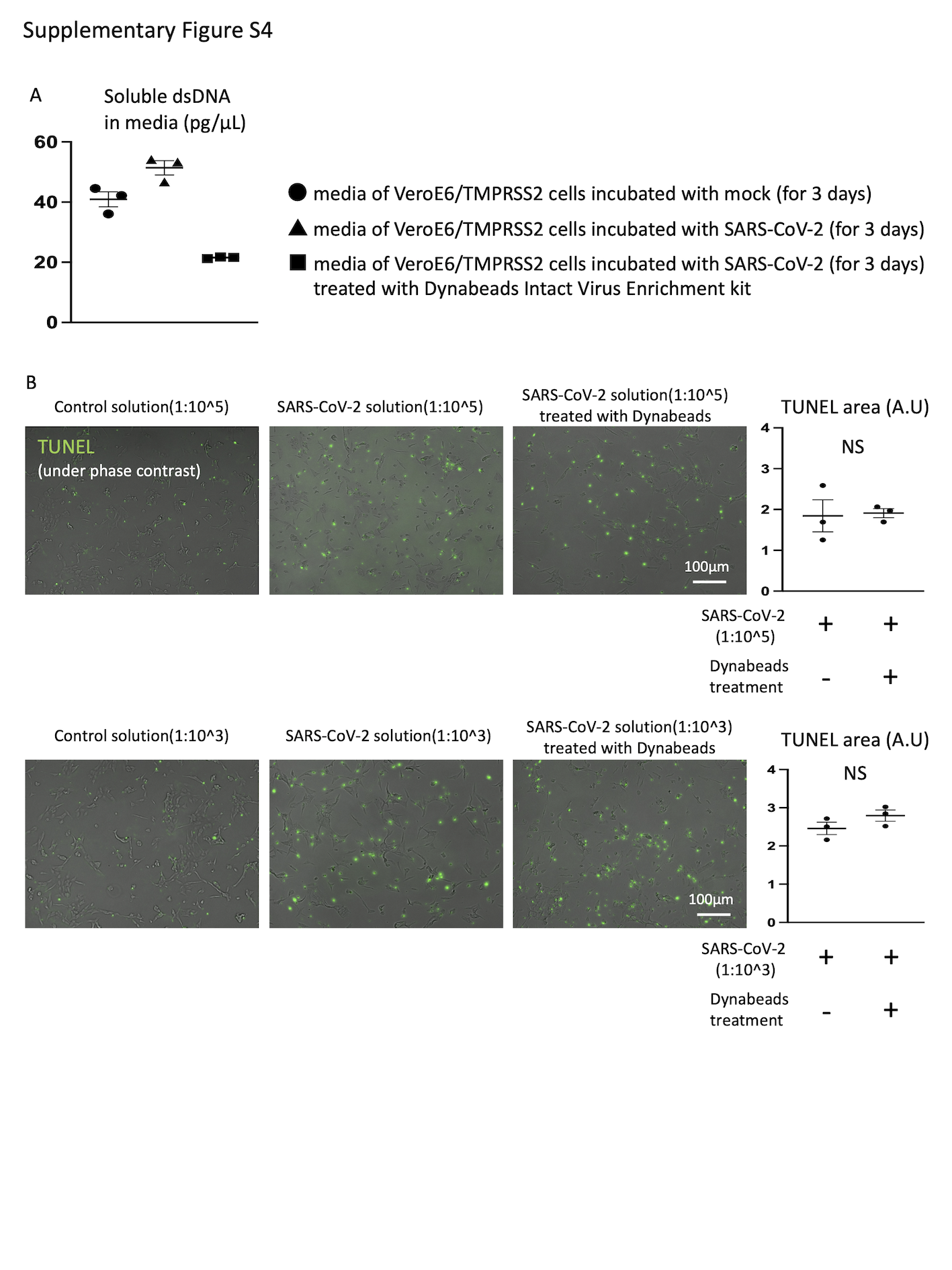

Supplement: Supplementary file 7 — Supplementary Figure S4 [file 41420_2023_1584_MOESM7_ESM.tif]

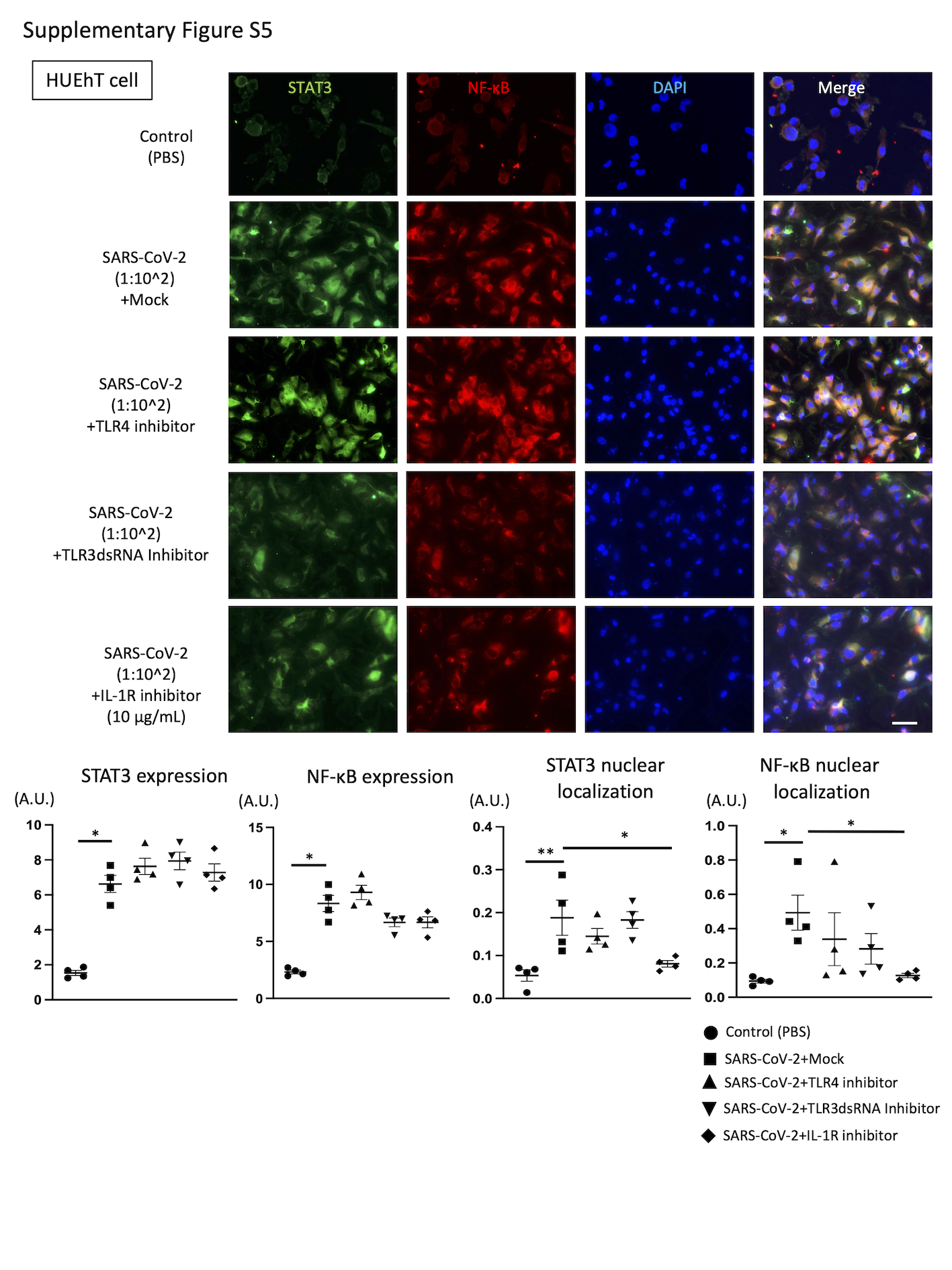

Supplement: Supplementary file 8 — Supplementary Figure S5 [file 41420_2023_1584_MOESM8_ESM.tif]

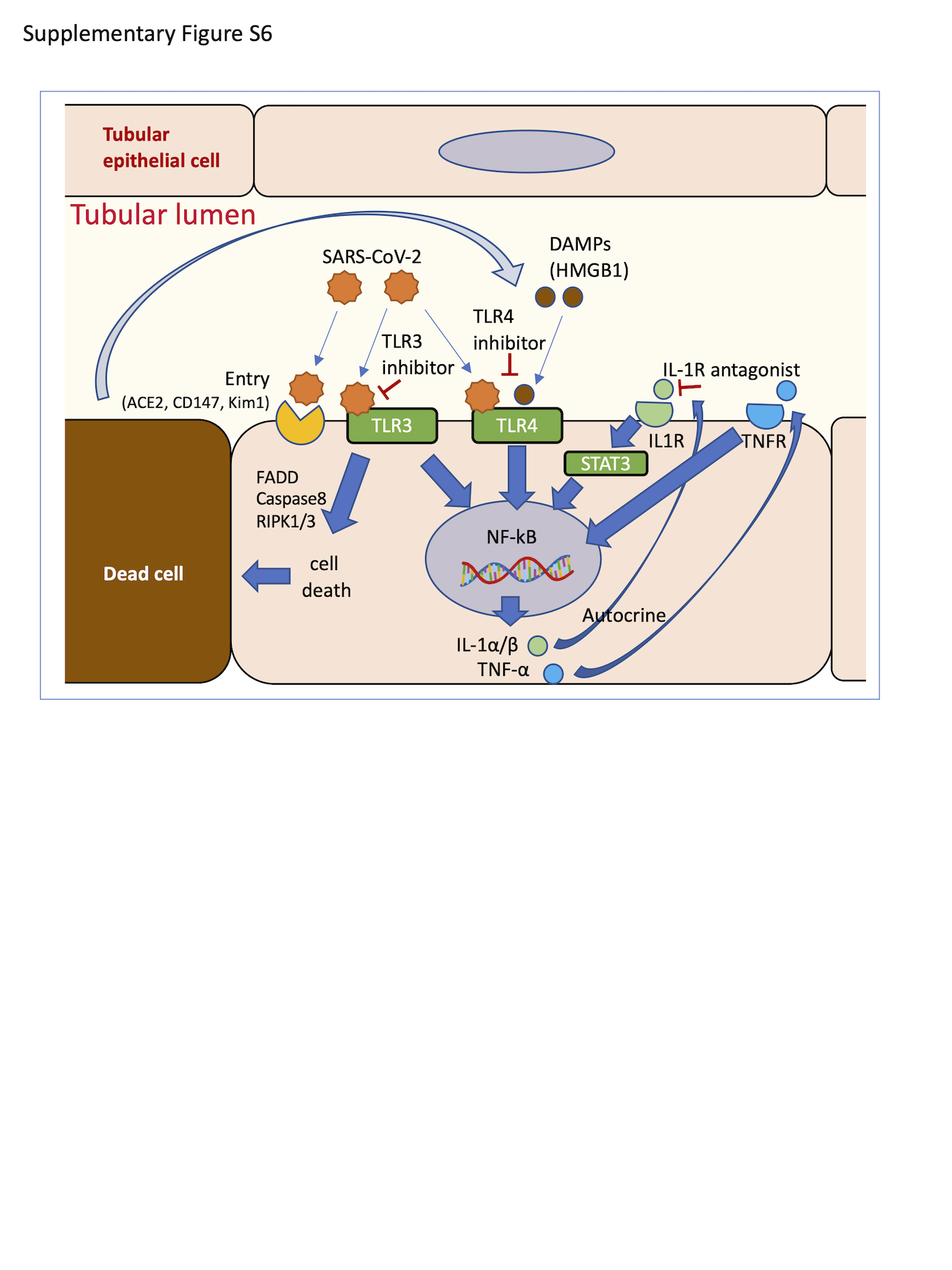

Supplement: Supplementary file 9 — Supplementary Figure S6 [file 41420_2023_1584_MOESM9_ESM.tif]
